# Toxic acute hepatitis and renal failure related to glue sniffing: a case report

**DOI:** 10.11604/pamj.2024.49.90.44057

**Published:** 2024-11-25

**Authors:** Meriam Khadhar, Ikram Mami, Hiba Ghabi, Azza Fekih, Syrine Tlili, Lamia Rais, Fethi Ben Hmida, Mohamed Karim Zouaghi

**Affiliations:** 1 Department of Nephrology, Dialysis and Kidney Transplantation, Mongi Slim Hospital, Tunis, Tunisia,; 2 Faculty of Medicine, University of Tunis El Manar, Tunis, Tunisia,; 3 Laboratory of Kidney Disease (LR00SP01) Tunis, Tunisia,; 4 Department of Nephrology, Dialysis and Kidney Transplantation, La Rabta Hospital, Tunis, Tunisia

**Keywords:** Glue sniffing, toxic nephropathy, acute kidney injury, hepatitis, case report

## Abstract

Glue sniffing is the most prevalent form of inhalant abuse worldwide, especially prominent in developing countries. Toluene, a solvent in glue, is identified as its primary toxic component. Chronic abuse leads to symptoms like muscle weakness, gastrointestinal problems, and central nervous system impairment. Acute complications such as kidney injury and hepatitis with cholestasis are rare but severe. This report details a 19-year-old male who presented with abdominal pain, respiratory distress, and hemoptysis after glue inhalation. He rapidly developed renal failure and hepatic damage. Renal biopsy confirmed acute tubular epithelial injury. Hemodialysis was initiated due to severe toxicity, with complete recovery of renal and hepatic functions achieved within 25 days. These findings underscore the need to consider toluene inhalation in the differential diagnosis of acute renal failure, especially in young individuals. Additionally, a brief review of the literature highlights the acute renal and hepatic toxicities associated with toluene abuse.

## Introduction

Glue sniffing is the most frequently abused inhalant drug, with toluene as the toxic component, leading to various complications upon abuse [1]. Reports commonly cite involvement of the central nervous system, gastrointestinal system, and muscular system [1]. Nevertheless, instances of acute kidney injury and hepatitis with cholestasis attributable to toluene abuse are rarely documented [2,3]. We report a case of rapidly progressive renal failure and hepatic impairment resulting from the inhalation of glue and provide a brief overview of the existing literature regarding the hepato-renal toxicity of toluene.

## Patient and observation

**Patient information:** a 19-year-old man presented to the emergency department with symptoms of asthenia and abdominal pain after inhaling glue. He was a healthy man and was not taking any oral medications. He was a current smoker but he had no history of alcohol consumption or allergies. On admission, the patient was conscious and eupneic. His blood pressure was 120/70 mmHg, with a heart rate of 85 bpm. The physical examination revealed a flat and soft abdomen without tenderness. His serum creatinine was 360 µmol/l on day one and then increased to 572 µmol/l on day two. Serum sodium (130 mmol/L) and serum chloride (96 mmol/l) were within the reference ranges, but serum potassium (5.4 mmol/l) was elevated. His peripheral blood cell counts were as follows: white blood cells (WBCs), 16.680 cells/mm^3^; hemoglobin, 14.7 g/dL; and platelets, 160.000 cells/mm^3^. The patient was referred to our nephrology department for the management of rapidly progressive renal failure.

**Clinical findings:** during the initial physical examination, we discovered that he had experienced a single episode of hemoptysis. The physical findings upon admission were as follows: height, 160 cm; weight, 62 kg; body mass index, 24.2 kg/m^2^; blood pressure, 130/70 mmHg; heart rate, 115/min, regular; respiratory rate, 24/min; and SpO_2_, 98% (O_2_, 15 L). Coarse crackles were heard in both lung fields. The patient was orthopneic but conscious and cooperative (Glasgow score 15/15).

**Figure 1:**
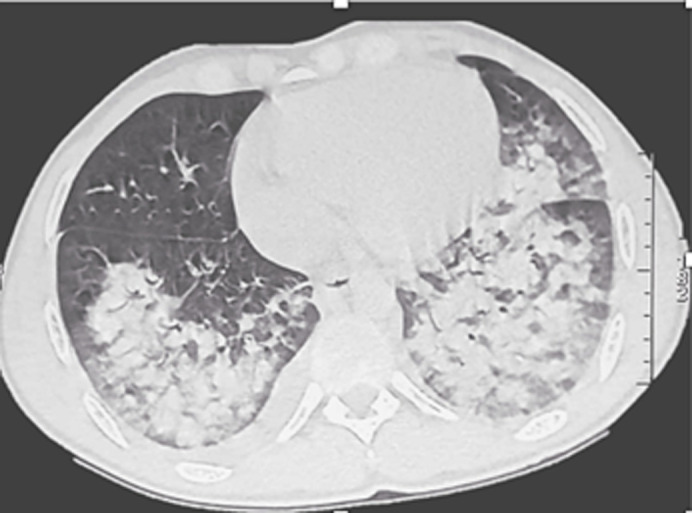
thoracic computed tomography showing bilateral parenchymal condensations with predominantly central ground glass opacities and bilateral pleural effusion

**Diagnostic assessment:** on admission, the patient's serum creatinine levels continued to rise (762 µmol/L). Other laboratory abnormalities included hyponatremia at 124 mmol/L, thrombocytopenia (127,000 platelets/mm^3^), elevated lactate dehydrogenase level (1152 U/L), hepatic cytolysis (ALT=258 U/L, AST=116 U/L), cholestasis (GGT=250 U/L), a low prothrombin count (PT 52%, INR: 1.6), and a significant inflammatory response (C-reactive protein at 94.6 mg/dL and white blood cells at 18.320/mm^3^). Lipasemia was normal. Antinuclear antibodies, anti-double-stranded DNA antibodies, antibodies to glomerular basement membrane, and antineutrophil cytoplasmic autoantibodies were negative. Testing for HIV and hepatitis B and C viruses also yielded negative results. Additionally, all microbiological examinations, including urine and blood tests, showed negative results. Polymerase chain reaction testing for SARS-CoV-2 was also negative. On abdominal ultrasound, both kidneys were of normal size, with regular contours and good cortico-medullary differentiation. The pyelocaliceal cavities were not dilated. The liver and spleen appeared homogeneous and were of normal size. The chest CT scan revealed bilateral parenchymal condensations accompanied by predominantly central ground glass opacities. Bilateral pleural effusion was also noted (Figure 1).

**Figure 2:**
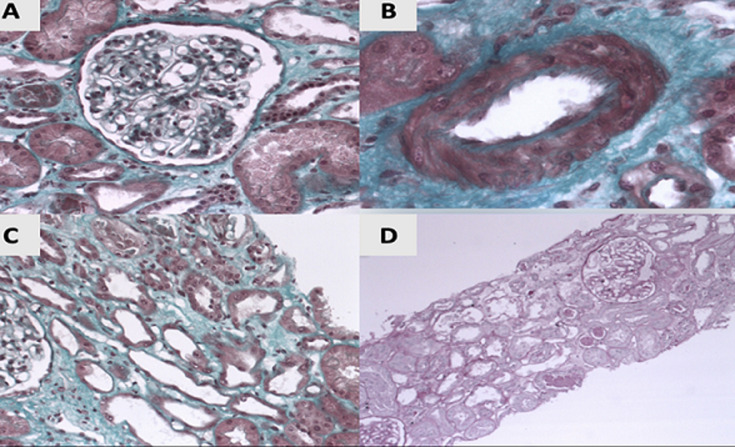
renal pathological findings: A) Masson’s trichrome (x200), optically normal glomerulus; B) Masson’s trichrome (x200), mild arteriolar hyalinosis; C) Masson’s trichrome (x200), diffuse acute tubular epithelial injury; D) periodic acid chiff (x100) diffuse interstitial fibroedema and acute tubular epithelial injury

**Diagnosis:** the patient has toxic hepato-nephritis. He was placed on oxygen (3l/min) and antibiotic therapy with cefotaxime and ofloxacin. An emergency dialysis depletion session was performed, which resulted in an improvement in the patient's respiratory condition post-dialysis. Four sessions of dialysis were required, and the patient showed a favorable progression. A kidney biopsy was performed 6 days after admission (15 days after inhalation of glue). Fourteen glomeruli were sampled for light microscopy and showed normal morphology. Diffuse acute tubular epithelial injury was observed, characterized by epithelial flattening, loss of brush border, and cellular desquamations in the lumens. There was diffuse interstitial fibroedema without interstitial infiltrate. Three small-caliber interlobular arteries were the site of multiple mycocytary vacuoles. Mild arteriolar hyalinosis was also present. No crystals were visible in polarized light (Figure 2). Immunofluorescence analysis for immunoglobulin A (IgA), IgG, IgM, kappa (κ) and lambda (λ) light chain, C3, C1q, fibrin, and albumin did not reveal any specific glomerular staining. However, segmental linear staining along the tubular basement membranes was observed with IgG and κ light chain, with a strength of 1+ on a scale of 0 to 3+. Together, these findings were compatible with a diagnosis of toxic hepato-nephritis with acute tubular injury. As our patient had been sniffing glue for 6 months, and no other etiologies were identified, the glue was incriminated as the cause of these disturbances. Unfortunately, we did not conduct a blood assay for toluene or the urinary hippurate test.

**Therapeutic interventions:** the patient required a total of four dialysis sessions and antibiotic therapy with cefotaxime and ofloxacin.

**Follow-up and outcome of interventions:** at dialysis cessation, the serum creatinine level decreased to 586 μmol/l. At the latest follow-up evaluation, conducted 20 days after the completion of hemodialysis, the serum creatinine level was 90 μmol/l and the hepatic assessment was within normal range.

**Patient perspective:** as a 19-year-old boy, my life took an unexpected turn when I found myself battling severe health complications due to glue sniffing. Like many others around the world, I turned to inhalant abuse as a means of seeking temporary relief or escape from life's challenges. Little did I know the grave dangers that lurked within the very substance I was using. Glue sniffing, particularly involving toluene, is alarmingly prevalent, especially in developing countries. Toluene, a solvent found in glue, is known to be highly toxic, yet its potential for causing acute kidney injury and hepatitis with cholestasis is often overlooked or underestimated. My journey began with seemingly innocuous symptoms - abdominal pain, respiratory distress, and a frightening episode of hemoptysis. However, these warning signs quickly escalated into a life-threatening crisis as I developed rapidly progressive renal failure and hepatic damage. A renal biopsy confirmed the extent of the damage, revealing diffuse acute tubular epithelial injury - a sobering realization of the havoc wreaked by inhalant abuse on my body. The gravity of my situation became apparent as hemodialysis was initiated to address the severe renal toxicity and respiratory distress.

It was a challenging time, filled with uncertainty and fear. However, through the dedicated care of healthcare professionals and the unwavering support of my loved ones, I began to see glimmers of hope. Remarkably, within 25 days of intensive treatment and support, I experienced a complete recovery of renal and hepatic functions. It was a miraculous turnaround that I am eternally grateful for - a second chance at life that I do not take lightly. Reflecting on my journey, I am compelled to share my story - not only as a cautionary tale but also as a beacon of hope. My experience underscores the critical need for heightened awareness surrounding the dangers of inhalant abuse, particularly among young individuals. Toluene inhalation must be considered in the differential diagnosis of acute unexplained renal failure, and comprehensive management strategies must be implemented to address the systemic effects of glue sniffing. Through education, advocacy, and support, we can work together to prevent others from experiencing the devastating consequences of inhalant abuse. My journey may have been fraught with challenges, but it has also instilled in me a renewed sense of purpose - to raise awareness, to support those in need, and to advocate for a healthier, safer future for all

**Informed consent:** the patient has provided consent for the publication of this case report.

## Discussion

The abuse of glue and other solvents is becoming increasingly common, particularly in developing countries, due to various factors including easy accessibility and affordability [1]. Toluene is the primary toxic substance found in glue sniffing. Common complications associated with the inhalation of toluene include muscle weakness, gastrointestinal issues such as abdominal pain and hematemesis, as well as neuropsychiatric disorders like changes in mental state, abnormalities in the cerebellum, and peripheral neuropathy [1]. Acute kidney injury and hepatitis with cholestasis have rarely been attributed to toluene abuse [2,3]. We present the case of a 19-year-old man who developed acute kidney injury (AKI) without any abnormalities in urinary sediment or hypertension. He also experienced abdominal pain, liver damage, respiratory distress, and one episode of hemoptysis 15 days after inhaling glue. We diagnosed toxic hepato-nephritis with acute tubular injury in a renal biopsy. We suspect that his symptoms may be related to glue inhalation. A significant strength of this case report lies in its detailed documentation of severe clinical manifestations of hepato-renal toxicity resulting from inhalation of glue containing toluene. This contributes significantly to the medical literature by providing a clear example of the potentially serious complications of inhalant abuse in young adults. However, a notable limitation is the absence of direct analyses for toluene in blood or urinary hippurate tests, which could have further bolstered the causal association between toluene exposure and the observed kidney and liver damage.

The reported renal manifestations in the literature include abnormal urine sediment with normal renal function, Fanconi syndrome with distal renal tubular acidosis (RTA) [4], rhabdomyolysis [5], anti-glomerular basement membrane antibody-mediated glomerulonephritis [3], acute and chronic renal failure [6] and nephrolithiasis [7]. In Table 1 summarizes previously reported cases by describing the main presentation, peak creatinine level, renal biopsy findings, the need for renal replacement therapy, and outcomes [1,2,4-9]. Exposure to toluene and its metabolite, hippuric acid, leads to renal impairments. Although toluene primarily affects the urinary system, it's crucial to highlight that serum creatinine levels increase in only about 20% of patients [10]. In addition to renal involvement, toluene abuse has been associated with liver damage, marked by increased levels of transaminases and ALP (alkaline phosphatase) [2,3]. In 1971, O'Brien *et al*. [2] reported a case of a glue sniffer who presented with severe hepatorenal toxicity and persistently elevated ALP levels even after 7 days of supportive treatment. Furthermore, Camara-Lemarroy *et al*. [10] documented that there was no increase in ALP levels, and GGT was only slightly elevated in patients who did not survive. Yurtseven *et al*. [1] described that even though ALP and GGT levels were within the normal range, the presence of jaundice, indicative of hepatobiliary injury, along with elevated transaminases, provided evidence of hepatotoxicity. However, hemodialysis was initiated due to severe renal toxicity and respiratory distress, as a suspected treatment for toluene poisoning of our patient. When dealing with severe toluene toxicity, hemodiafiltration (HDF) should be prioritized as the primary treatment method. This is attributed to its safe technique, ability to provide controlled ultrafiltration, and more effective elimination of toxic metabolites using smaller dialysis filters compared to hemodialysis and plasma exchange [1]. Unfortunately, HDF was not available in our department.

**Table 1:** renal failure and hepatitis related to glue sniffing: summary of literature

Authors Year	Gender	Age	Practice of Glue sniffing	Main presentation	Peak creatinine level	Renal biopsy	Need for RRT	Outcomes
O'Brien *et al*. /1971	M	19 years	3 years	Hepatorenal damage	7 mg/100ml	Not mentioned	Yes PD	The jaundice gradually disappeared Renal function returned to normal
Taher *et al*. /1974	M	23 years	3 years	Distal renal tubular acidosis	1.6 mg/100ml	Not mentioned	No	Reversible distal renal tubular acidosis
Reisin *et al*. /1975	M	49 years	Not mentioned	Acute renal failure due to rhabdomyolysis	548 umol/l	Not mentioned	Yes	Six months later: serum creatinine was 88.4 umol/l and normal urinalysis
Moss *et al*. /1980	F	27 years	9 months	Fanconi syndrome with distal renal tubular acidosis	Not mentioned	Not mentioned	No	Correction of the serum abnormalities particularly hyperchloremic acidosis
Kroeger *et al*. /1980	M	23 years	5 years	Stone formation	Not Mentioned	Glomerular basement membrane thickness /instances of fine splitting of the membrane. IF: IgG, IgM, IgA, complement, fibrin and fibrinogen were negative	No	Four kidney stone operations in 15 months
Will *et al*. /1981	M	14 years	2 years and 8 months	Acute renal failure	1493 mg/100 ml	Not mentioned	No	Urea and creatinine concentrations remained normal, microscopic hematuria persisted after six weeks' abstinence from glue
Russ *et al*. /1981	M	20 years	9 months	Acute renal failure	Not Mentioned	Tubular atrophy and interstitial fibrosis	Not mentioned	Irreversible acute renal failure
Yurteseven *et al*. /2018	M	16 years	8 months	Anuric renal failure and hepatitis	9.6 mg/dl	Not mentioned	Yes HDF	Day 7: blood urea and serum creatinine levels were within normal limits with normal urine output Day 10: jaundice gradually disappeared and hepatic functions returned to normal
Our case /2024	M	19 years	6 months	Renal failure and hepatitis	779 umol/l	Tubular necrosis/ multiple mycocyte vacuoles	Yes HD	Day 25: serum creatinine level was 90 μmol/l and the hepatic assessment was within normal range

M: Male; F: Female; RRT: renal replacement therapy; PD; Peritoneal dialysis; HDF: hemodiafiltration; HD: hemodialysis; IF: Immunofluorescence

## Conclusion

In summary, this case report illustrates a severe instance of hepato-renal toxicity following glue inhalation, emphasizing the grave consequences of toluene abuse among young individuals. The detailed clinical documentation enriches our understanding of the detrimental effects of inhalant abuse, highlighting acute kidney injury and hepatitis with cholestasis as rare but serious complications. Despite the limitations, such as the absence of direct toluene analyses, this report underscores the critical need for heightened awareness and early recognition of toluene inhalation in cases of unexplained renal and hepatic failure. Comprehensive management strategies and preventive measures are imperative to mitigate the significant health risks associated with inhalant abuse.
